# Histone Deacetylation Inhibitors as Modulators of Regulatory T Cells

**DOI:** 10.3390/ijms21072356

**Published:** 2020-03-29

**Authors:** Andreas von Knethen, Ulrike Heinicke, Andreas Weigert, Kai Zacharowski, Bernhard Brüne

**Affiliations:** 1 Department of Anaesthesiology, Intensive Care Medicine and Pain Therapy, University Hospital Frankfurt, 60590 Frankfurt, Germany; ulrike.heinicke@kgu.de (U.H.); kai.zacharowski@kgu.de (K.Z.); 2 Fraunhofer—IME, Project Group Translational Medicine and Pharmacology (TMP), 60596 Frankfurt, Germany; b.bruene@biochem.uni-frankfurt.de; 3 Institute of Biochemistry I, Faculty of Medicine, Goethe-University Frankfurt/Main, 60590 Frankfurt, Germany; weigert@biochem.uni-frankfurt.de

**Keywords:** tolerance induction, epigenetics, Foxp3 expression, histone deacetylase inhibitor, sepsis, transplantation, autoimmunity, Treg

## Abstract

Regulatory T cells (T_regs_) are important mediators of immunological self-tolerance and homeostasis. Being cluster of differentiation 4^+^Forkhead box protein3^+^ (CD4^+^FOXP3^+^), these cells are a subset of CD4^+^ T lymphocytes and can originate from the thymus (tT_regs_) or from the periphery (pT_regs_). The malfunction of CD4^+^ T_regs_ is associated with autoimmune responses such as rheumatoid arthritis (RA), multiple sclerosis (MS), type 1 diabetes (T1D), inflammatory bowel diseases (IBD), psoriasis, systemic lupus erythematosus (SLE), and transplant rejection. Recent evidence supports an opposed role in sepsis. Therefore, maintaining functional T_regs_ is considered as a therapy regimen to prevent autoimmunity and allograft rejection, whereas blocking T_reg_ differentiation might be favorable in sepsis patients. It has been shown that T_regs_ can be generated from conventional naïve T cells, called iT_regs_, due to their induced differentiation. Moreover, T_regs_ can be effectively expanded in vitro based on blood-derived tT_regs_. Taking into consideration that the suppressive role of T_regs_ has been mainly attributed to the expression and function of the transcription factor Foxp3, modulating its expression and binding to the promoter regions of target genes by altering the chromatin histone acetylation state may turn out beneficial. Hence, we discuss the role of histone deacetylation inhibitors as epigenetic modulators of T_regs_ in this review in detail.

## 1. Introduction

Regulatory T cells (T_regs_) are important to guarantee immunological self-tolerance and homeostasis. Since their first description in 1995 [1], several subpopulations of T_regs_ have been described to fulfill these requirements [2]. First, T_regs_ can originate from the thymus. Accordingly, these T_regs_ are named tT_regs_. [3]. Second, T_regs_ can develop from effector T cells in the periphery and are thus designated as pT_regs_ [4]. This usually happens upon the activation of post-naïve effectors with mainly oral antigens in the presences of specific cytokines. In the thymus, thymocytes are educated to self-antigenic peptides first in the cortex and then in the medulla with medium affinity, whereas thymocytes destined to become T_regs_ are educated to recognized self-antigenic peptides with high affinity, mainly in the cortical–medullary junction and the medulla, before being released to the periphery. To achieve this, self-reactive thymocytes are eliminated by negative selection [5]. However, although this mechanism is very effective, some self-reactive, and thus possibly autoimmunity-inducing T cells, escape this machinery [6,7]. Therefore, a system must exist that restricts activity of these cells. This was proven by the classical thymectomy experiment in neonate mice, which showed T cell-dependent autoimmunity when the thymus was removed at day three after birth but not at day one or day seven [8,9,10]. These tT_regs_, which migrate to the periphery after day three, are essential for self-tolerance. Recent evidence identified thymocyte apoptosis, occurring after birth [11], as leading to the intrathymic release of transforming growth factor (TGF)-β as reason for the delayed tT_regs_ export compared to cluster of differentiation (CD)4^+^ single positive (SP) thymocytes [12]. TGF-β initiates Foxp3 expression and tT_regs_ development [12]. However, earlier data have shown normal tT_regs_ development in mice deficient for TGF-β 1 but significantly reduced pT_regs_ [13]. The expression of the transcription factor Foxp3 is a marker of T_regs_. The activation of the corresponding gene locus is a multistep process [14,15]. It requires a high affinity binding of major histocompatibility complex (MHC)-self peptide complexes from thymic antigen-presenting cells (APCs) to the T cell receptor (TCR) and costimulatory signals as well as cytokine environments (IL-2) [16,17]. Foxp3 provokes the expression of target genes, which are important to trigger and maintain the immune suppressive T_regs_ phenotype, as shown by genome-wide analyses in mice and humans [18,19]. In mice, Foxp3 binding results in both the activation and repression of its target genes. This was determined by chromatin immunoprecipitation (ChIP) against epigenetic markers such as acetylated H3K9/14 (AcH3), tri-methyl H3K4 (Me3K4), and tri-methyl H3K27 (Me3K27). These data identified the cell surface molecules Il2ra (CD25), Ctla4 (CD152), Nt5e (CD73), and Icos (CD278) as well as the transcription factor Ikzf2 (Helios) to be Foxp3-dependently upregulated based on the chromatin markers AcH3 and Me3K4. In contrast, the phosphodiesterase Pde3b showed tri-methylation at H3K27, mandatory for its inhibited expression [20]. For the latter one, it was recently shown that Foxp3 also induces the microRNA-142-5p, which as an intracellular cAMP sensor leads to the posttranscriptional repression of the cAMP hydrolyzing enzyme Pde3b [21]. In human T_regs_, a similar expression profile was observed, demonstrating the selective gene expression of IKZF2 (HELIOS) [22], IL2RA (CD25), and cytotoxic T lymphocyte associated protein 4 (CTLA4) (CD152) [23]. Moreover, the expression of the T cell survival factor IL-2 is Foxp3-dependently downregulated [24]. Further proof for the significance of Foxp3 in T_reg_ differentiation and function came from studies analyzing mutations associated with the nonfunctional expression of Foxp3 in humans, causing IPEX syndrome (immune dysregulation polyendocrinopathy and enteropathy), which requires bone marrow transplantation in early childhood [25]. In mice, a lack of Foxp3 expression, as observed in scurfy mice, induces a similar phenotype [26]. The role of Foxp3 has been further corroborated in mice where the experimental depletion of Foxp3^+^ T_regs_ in healthy adult mice has been found to provoke autoimmunity and death [26,27].

Considering this important role of T_regs_ in maintaining immune self-tolerance, treatment with in vitro generated T_regs_ may be a therapeutic approach towards autoimmune-mediated diseases. T_regs_ can be generated ex vivo from conventional naïve T cells after TCR stimulation in the presence of TGF-β and IL-2. These cells are named iT_regs_ according to their induced differentiation [28]. However, the stability of Foxp3 expression in these cells is much lower compared to tT_regs_ or pT_regs_ [29]. Therefore, treatment regimens to prolong and stabilize Foxp3 expression in iT_regs_ are a topic of current research. In contrast, the role of T_regs_ in sepsis patients seems to be deleterious [30,31,32,33,34]. In this case, reducing the pT_reg_ or tT_reg_ number and function might prevent immunosuppression, consequently improving survival. Thus, epigenetic modifications are an interesting approach to cope with these opposite tasks.

## 2. Role of T_regs_ in Disease

### 2.1. Autoimmune Diseases

Based on T_regs’_ role on the induction and maintenance of peripheral tolerance, T_reg_ dysfunction is associated with severe autoimmune conditions. Disease patterns such as systemic lupus erythematosus (SLE) [35] and organ-specific autoimmune diseases, e.g., type 1 diabetes (T1D) [36] and psoriasis [37], have been attributed to a reduced number of T_regs_ or the failure of their function. Considering this important role of T_regs_, therapies have been developed to medicate autoimmune disorders (for reviews of single clinical trials, see [38,39]). Phase I clinical studies have been using autologous T_regs_ to treat SLE, pemphigus vulgaris, or T1D. Moreover, already published studies have supported an ameliorative impact of these treatment regimens for T1D, prolonging the survival of β -cells [40,41]. Beside these polyclonal T_reg_ therapies, T_reg_-enhancing drugs are of interest. Among others, rapamycin-dependent mTOR inhibition was used to efficiently expand human T_reg_ cells [42] and to treat SLE patients, with significant improvement of the clinical outcome [43]. Several other approaches have been shown to block mTOR activation in animal models and T cells derived from the blood of SLE patients, including the blocking of S1P receptors, antioxidants, and calmodulin kinase type II and type IV inhibitors [44,45,46,47]. Low-dose IL-2 treatment has also been identified for the treatment of patients with diseases associated with a decreased number of T_regs_ such as SLE [48,49,50]. Considering that IL-2 activates T_regs_ as well as T_eff_, a dose finding study was performed [51]. In this setting, clinical phase II trials are already running for the treatment of rheumatoid arthritis (RA), SLE, multiple sclerosis (MS), T1D, and amyotrophic lateral sclerosis (ALS). 

### 2.2. Transplant Rejection

Patients with end-stage organ failure need organ transplantation as their therapy of choice. Moreover, an autologous bone marrow transfer is required in patients suffering from chemotherapeutic treatment or an allogenic transfer in patients with lymphoma or leukemia. As expected, T_regs_ are important mediators of graft tolerance induction following these transplantations. Based on their immunosuppressive function, an increased number of T_regs_ in the periphery and graft microenvironment has been attributed to confer graft tolerance, thus guaranteeing a long lasting life of solid organ transplants or transferred bone marrow [39,52,53]. Considering this important role of T_regs_, in-man phase 1 and phase 2 clinical trials, as summarized in [53,54], have been performed [55,56,57] or registered. Briefly, liver transplantations were supported with T_reg_ cell therapy, following T_reg_ generation using autologous T_regs_ stimulated with irradiated donor PBMCs with inhibited costimulation, autologous donor antigen-expanded T_regs_, or autologous, polyclonally-expanded T_regs_. A similar setup was used to T_reg_-dependently assist liver transplantation. Additionally, in bone marrow transfer approaches, T_regs_ have been shown to suppress T cell alloreactions and to prevent graft-versus-host disease in mouse models [28,58,59,60] and in the human situation [61].

### 2.3. Sepsis

Sepsis is a syndrome where T cell depletion and, consequently, an inappropriate immune response to the recurring initial infection or acquired second infection is one characteristic [62]. Consequently, it is of interest to follow the fate of T_regs_ during sepsis initiation and progression. As shown recently by Carvelli et al. [32], the number of T_regs_ was decreased in patients with septic shock, which is in some discrepancy to previous reports that have shown an increased number of T_reg_ cells in these patients as one reason for long-term immune-suppression [33,34]. Based on these contradictory data, the role of T_regs_ in sepsis needs further evaluation. Thus, the different stages, i.e., infection, organ dysfunction caused by an inappropriate immune response, septic shock, and finally sepsis survivors, must be carefully examined to draw any conclusion whether T_regs_ are important to block an overwhelming immune response or whether these cells are crucial for the resolution of inflammation. In both situations, excessive T_regs_ might be detrimental. Therefore, the pharmaceutical or immunological fine tuning of T_regs_ will be advantageous to intervene with the respective prevailing pro- vs. anti-inflammatory responses. This has also been shown for the role of T_regs_ in resolving lung injury [63]. In this animal approach, mice were treated with lipopolysaccharide (LPS) or recombinant high-mobility-group-protein B1 (HMGB1), which is a key mediator during inflammation to induce acute lung injury (ALI). T_regs_ were modulated with myeloid-specific β-catenin and phosphatase and tensin homologue (PTEN)-knockout mice. As shown in Table 1, several rodent studies have shown an altered number of T_regs_ following polymicrobial sepsis by cecal ligation and puncture (CLP). In these studies, various mouse (BALB/c, C57BL/6, FVB/N, ICR, NMRI) and rat strains (Fischer, Wistar, Wistar Hannover, Sprague Dawley) have been used, applying different severities of polymicrobial sepsis. This can be achieved by the diameter of the needle and the number of cecum perforations [64,65]. Most studies have demonstrated an increase of T_regs_ in spleen or blood, independently from the execution of the model. This is in line with the assumption that T_regs_ are generally involved in downregulating the immune response, thus contributing to an immunosuppressed phenotype during sepsis. Interestingly, our own data support this notion. We found that a prevention of CLP-dependent liver damage was associated with a decreased number of liver localized T_regs_ [66]. Thus, reducing the T_regs_ count might be a prerequisite for improving sepsis outcome by restoring a functional T cell response.

## 3. HAT and HDAC Activities in T_reg_ Differentiation

Epigenetic, i.e., reversible modifications of chromatin that do not alter the DNA sequence, can be achieved by inhibiting histone deacetylase (HDACs) to maintain chromatin histone acetylation, consequently keeping genomic DNA accessible for the binding of transcription factors and the RNA polymerase. This finally allows for the increased expression of genes known to be involved in T_reg_ differentiation and function. 

### 3.1. Foxp3

**Figure 1:**
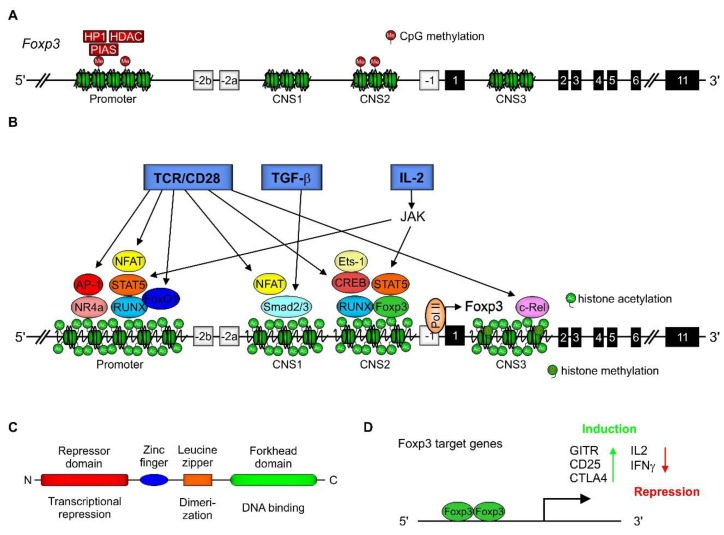
Foxp3 gene expression in T_regs_. (**A**) The gene structure of mouse Foxp3 in naïve cluster of differentiation (CD)4^+^ T cells. The Foxp3 gene contains five regulatory elements. 5′ starting with one out of four conserved non-coding sequences (CNS0-3). The CNS0 site has recently been identified as a super-enhancer, bound by special AT-rich sequence-binding protein 1 (SATB1), being responsible for T_reg_-lineage-specific gene expression. Between CNS0 and CNS1 the Foxp3 promoter region is located. The binding of the SUMO E3 ligase protein inhibitor of activated signal transducer and activator of transcription (STAT) (PIAS)1 to the promoter region enables the tying of methyltransferases and heterochromatin protein 1 (HP1) to this site, maintaining the Foxp3 gene in a methylated and inactive, so called condensed, state. The methylation of the CNS2 region is also contributing to this heterochromatin structure. The Foxp3 gene contains 11 translated exons, encoding a protein of 431 amino acids in humans and 429 amino acids in mice. (**B**) The induction of Foxp3 expression in T_regs_ is initiated by the binding of self-antigens to the T cell receptor (TCR) in combination with a co-stimulatory signal such as CD28. Moreover, transforming growth factor (TGF)-β and IL-2 are essential for effective Foxp3 gene transcription. These three activation signals provoke the recruitment of nuclear factor of activated T cells (NFAT), activator protein 1 (AP-1), STAT5, FoxO1, Runt-related transcription factor 1 (RUNX), and nuclear receptor 4a (NR4a) to the promoter region, NFAT and SMAD2/3 to CNS1, Ets1, cAMP-responsive element binding protein (CREB), STAT5, RUNX, and Foxp3 to CNS2, and finally c-Rel to the CNS3 site. Additionally, Smad4 is required for Foxp3 expression. Moreover, retinoid x receptor/retinoid acid receptor (RAR/RXR) heterodimers enhance Foxp3 expression following retinoid acid stimulation, whereas STAT3 is important for Foxp3 downregulation. (**C**) Domain structure of Foxp3. (**D**) Foxp3 target genes.

Foxp3 is a member of the Forkhead box protein (Foxp) subfamily of transcription factors. Due to its function as a master regulator of tT_reg_ and pT_reg_ differentiation and immunosuppressive performance, understanding Foxp3’s transcriptional, translational, and post-translational regulation is important [14,98,99]. Therefore first, the gene structure is crucial [100,101]. The expression of the Foxp3 gene is mediated by five control elements. Starting 5′, the first conserved non-coding sequence (CNS) 0 [102], which is the binding site for the special AT-rich sequence-binding protein (SATB) 1, a super-enhancer that enables T_reg_-lineage-specific gene induction was recently identified [102,103]. Following CNS0, the promoter region [104,105] and three further CNS (CNS1–3) are located. CNS1, located in intron 1, is associated with TGF-β inducibility (TGF-β sensor) [105]. CNS2, also located in intron 1, is the so called T_reg_-cell-specific demethylation region (TSDR) [106,107] and CNS3, localized in intron 3, named the Foxp3 pioneer element, is known to confer the NF-κB inducibility of Foxp3 [108]. All these five elements are mainly characterized by the existence of CpG islands (promoter, CNS2) and histones, which can be acetylated (promoter, CNS0-3) or show permissive methylation (CNS3) [18,102]. Thus, these regulatory structures are targets for epigenetic modifications, altering the accessibility of the Foxp3 gene [109]. In naïve CD4^+^ T cells, CpGs are heavily methylated (Figure 1A), silencing the *Foxp3* gene [18]. Especially, the promoter region is the target of protein inhibitor of activated STAT (signal transducer and activator of transcription) (PIAS1), a SUMO E3 ligase, which restricts T_reg_ differentiation by recruiting DNA methyltransferases and heterochromatin protein 1 to the Foxp3 promoter [110]. Following DNA demethylation, histone acetylation, and permissive methylation, Foxp3 expression and, consequently, T_reg_ differentiation are induced by the activation of transcription factors in response to T cell receptor (TCR) engagement, CD28 co-stimulation, IL-2 treatment, and TGF-β addition. Established transcription factors are activator protein 1 (AP-1) (promoter) [111], cAMP-responsive element binding protein (CREB) (CNS2) [105], Ets-1 (CNS2) [112,113], FoxO1 (promoter) [114], nuclear factor of activated T cells (NFAT) (promoter, CNS1) [18,111], nuclear receptor 4a (NR4a) (promoter) [115], c-Rel (CNS3) [108], retinoid x receptor/retinoid acid receptor (RXR/RAR) (promoter, CNS1) [116,117], Runt-related transcription factor 1 (RUNX) (promoter, CNS2) [118], STAT3/5 (promoter, CNS2) [119], and SMAD2/3/4 (CNS1) [120,121] (Figure 1B).

**Figure 2:**
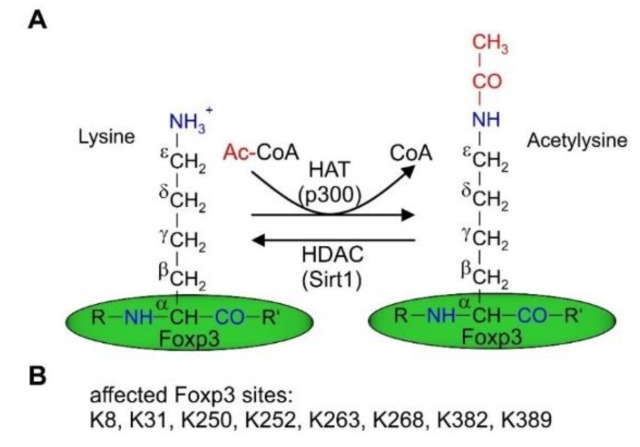
HAT and HDAC binding to Foxp3. (**A**) Amino groups located at the ε-CH2 group of lysines can be actetylated by histone acetylases (HATs) such as p300 or TI60 leading to acetylysine, which enhances Foxp3 stability by preventing its proteasomal degradation. Reciprocally histone deacetylases (HDACs) such as Sirt1 deacetylate lysines of Foxp3, which are acetylated at the amino-group next to the ε-C atom. Deacetylated Foxp3 is prone to proteasomal degradation [124]. (**B**) Lysines of Foxp3, which have been identified as targets for acetylation [122,125,132,133].

The Foxp3 protein contains four domains, including a repressor domain at the N-terminal end (responsible for transcriptional repression), a zinc finger domain with a so-far unclear function, a leucine zipper domain (important for dimerization), and, finally at its C-terminus, the Forkhead domain, which is important for DNA-binding (Figure 1C). It has been established that the repressor domain located at the N-terminus of Foxp3 is associated with the downregulation of the expression of HIF1α, RORγt, RORα, and Eos. Thus, among others, differentiation towards a Th17 phenotype is prevented [121]. When expressed, Foxp3 can form heterodimers with FoxO1, keeping Foxp3 in an inactive state. It can transiently homodimerize, which enables its regulation, or stably consequently leading to the expression or repression of target genes (Figure 1D). Moreover, transient Foxp3 homodimers may combine as clusters. Foxp3 can additionally bind roughly 700 different proteins, which is important to activate or repress the expression of target genes. Stable Foxp3 coiled-coil-mediated homodimerization is essential for T_reg_ function [122]. As shown in Figure 2A, Foxp3 associates with histone acetylases (HATs) (e.g., p300 or HIV-Tat-interactive protein (TIP60) [123]), leading to Foxp3 (hyper)acetylation, which increases Foxp3 stability as well as HDACs (e.g., silent information regulator 1 (SIRT1) or HDAC5 [124,125]), which reciprocally deacetylate Foxp3, making it more susceptible for proteasomal degradation [126]. Foxp3 lysine residues identified to be affected are shown in Figure 2B. Foxp3 expression and function are also regulated by the histone H3K27 methyltransferase enhancer of zeste homolog 2 (EZH2), which is not expressed in naïve T_regs_ and is upregulated in CD28-activated T_regs_, provoking a stable T_reg_ phenotype by allowing for Foxp3 expression and stabilization [127,128]. Consequently, EZH2-specific inhibitors as well as specifically disrupting EZH2 in T_regs_, reduced Foxp3 expression, and concomitantly attenuated the immune suppressive T_reg_ phenotype [127]. However, Foxp3 binding to EZH2 seems to inactivate Foxp3 [129]. Correspondingly, histone demethylases are involved in Foxp3 regulation. There, Jumonji domain-containing 3 (Jmjd3) is the most prominent demethylase, responsible for H3K27me2 and H3K4me3 demethylation, provoking Foxp3 expression and, accordingly, promoting T_reg_ differentiation [130]. Interestingly, in an acute lung injury (ALI) model in mice, the expression of JMJD3 is downregulated in T_regs_ isolated from the lungs [131]. These data support the notion of an organ and microenvironment specificity of T_regs_.

### 3.2. Cytotoxic T Lymphocyte-Associated Protein 4 (CTLA4 or CD152)

Considering a connection of the suppressive effect conferred by T_regs_ and their CTLA4 expression [134,135], mechanistic insights into the regulation of CTLA4 expression are important. CTLA4 is a co-inhibitor that is generally upregulated upon antigen stimulation via the TCR to prevent an uncontrolled immune response [136,137], allowing for the fine tuning or consequently shutdown of the immune response as an immune checkpoint [138]. This is achieved by its higher affinity to the co-activators CD80/86 (B7-1/-2) expressed on antigen-presenting cells such as macrophages (MΦ) and dendritic cells (DC) compared to the co-activator CD28. Besides this role, CTLA4 is constitutively expressed on T_regs_ [139,140], contributing to the immunosuppressive phenotype of these cells [141]. Moreover, it has been shown that the transgenic expression of CTLA4 is one prerequisite of converting a conventional to a regulatory T cell [142]. When expressed on the T cell surface, CTLA4 binds to CD80/CD86 on antigen-presenting cells (APC) with a higher affinity than CD28, downregulating CD80/86 on DC to decrease the potency of APC to activate T cells [135]. From this data, it is obvious to assume that altering CTLA4 expression, i.e., to enhance or downregulate its expression, will be an appropriate treatment regime in autoimmune diseases to enhance T_reg_-dependent tolerance induction, e.g., to prevent cardiac allograft rejection [143], or to inhibit this immune-suppressive reaction to enhance anti-tumor immunity, e.g., by using CTLA4-neutralizing antibodies [144]. The HDAC canonical pan-inhibitor SAHA (suberoylanilide hydroxamic acid, Vorinostat), inhibiting HDAC1-9 with similar potency, has been shown to enhance CTLA4 expression in T_regs_ [143]. This upregulation was even enhanced when tacrolimus was added to inhibit calcineurin in parallel. Additionally, SAHA promoted selectively effector T cell apoptosis, which is consequently associated with an increased T_reg_ proportion. This combined setting might be a therapeutic concept in preventing allograft rejection.

### 3.3. HDACs and HDACi as a Starting Point for Altering T_reg_ Function

Thus far, 18 HDAC enzymes have been described. Eleven are Zn^2+^-dependent (HDAC1-11) and seven need NAD^+^ (Sirt1-7) for their activity. Though there have already been several clinical trials using HDAC inhibitors (HDACi) for treatment in oncology, none have been initiated for the therapy of autoimmune diseases. Based on the use of HDACi to change the epigenetic structure of T_reg_-lineage-dependent genes, experiments using pan-HDACi have been performed. It has been shown that the differentiation of human CD25^high^Foxp3^+^ T_regs_ into IL-17 producing cells can be prevented by the HDACi trichostatin A (TSA) [145]. TSA inhibits, similarly to SAHA, HDAC1-9 without any preference [146]. Taking this unspecific inhibition into consideration, it is difficult to provide any data on the role of a single HDAC, apart from mouse knockout studies or HDACs, where specific inhibitors already are at hand. As shown in Table 2, T_regs_ express class I HDACs 1, 2, 3, and 8 [147], class IIa HDACs 5, 7, and 9 [148], class IIb HDACs 6 and 10 [149], unrelated class III SIRTs 1, 2, 3, and 4 [150], and class IV HDAC 11 [151]. 

According to the diverse roles of HDACs in T_reg_ immunology (see Graphical Abstract), corresponding HDAC inhibitors might be used to reduce or enhance T_reg_ cell number and function. Obviously, the expression of Foxp3 is an essential element in T_reg_ differentiation. Its transcription is inhibited by HDAC10 [167]. HDAC10 deletion in mice has been shown to enhance Foxp3 stability and increase H2K4Me3-activating marks on the Foxp3 promoter and CNS2 region [167]. Moreover, SIRT2 [171] and SIRT4 [173] downregulate protein Foxp3 expression by a so far unknown mechanism. Blocking these three HDACs will likely increase Foxp3 expression and concomitantly start and enhance T_reg_ differentiation. HDAC7 associates with NR4a and Foxp3, being involved in the Foxp3-dependent repression of target genes. Following protein kinase D-dependent phosphorylation, HDAC7 is exported from the nucleus, consequently allowing for gene expression [174]. HDAC3, 6, 9, 11, and SIRT1 have been established to deacetylate Foxp3, which target it for proteasomal degradation [155,158,160,175]. Moreover, HDAC9 inhibits the expression of PPARgamma coactivator 1 alpha (PGC1α), an important factor in inducing proteins of the oxidative phosphorylation (OXPHOS)-system, important for the mitochondrial-dependent energy supply of the cells [172]. Finally, HDAC1 has been attributed to block the activity of the transcription factor RUNX, mandatory to maintain CD4^+^ T cell integrity [152,176,177].

In various models, the role of HDAC inhibition or deletion has been determined. Briefly, the inhibition of class I HDACs in models of cardiac allograft transplantation (CAT) or colitis has shown an enhanced T_reg_ function following HDAC2 deletion, thus preventing HDAC2 association with Foxp3 [153,154], whereas the blockage of HDAC1, 3, and 8 has been shown to provoke an attenuated T_reg_ number and function by destabilizing Foxp3 [152,153,155,156]. Blocking class IIa HDACs attenuates T_reg_ function following HDAC5 and 7 inhibition in CAT and positive and negative selection in the thymus [157,158,159], whereas blocked HDAC9 enhances T_reg_ function by Foxp3 stabilization in a colitis model [132,160,161]. Interestingly, class IIb HDACs are only involved Foxp3 destabilization, as shown in cystic fibrosis, collagen-induced arthritis, juvenile idiopathic arthritis, and lupus prone mice, as well as in cardiac allograft transplantation and colitis. Thus, their inhibition enhances and restores T_reg_-dependent effects [160,162,163,164,165,166,167,168,169]. Members of the sirtuin-family of HDACs (class III HDACs) are important regulators of the inflammatory stress response in immune and non-immune cells linking inflammation and metabolism [178,179]. Therefore, their role in T_reg_ cell differentiation is mainly characterized by a Foxp3 destabilizing effect in murine sepsis (cecal-ligation and puncture), heterotrophic cardiac and orthotropic renal allograft transplantation, colitis [55,87,126,160,169,170,171,180], and by preventing Foxp3 expression in a mouse model of transient middle cerebral artery occlusion [173]. In contrast, SIRT3 is important for the metabolic adaption of T_regs_, which makes it necessary for their function. Thus, SIRT3 inhibition is associated with a reduced number of T_regs_ in cardiac allograft transplantation [172]. Lastly, intervening with the function of HDAC11, the only class IV HDAC, has been shown to result in Foxp3 stabilization, enhancing T_reg_ function in cardiac allograft transplantation [158].

## 4. Concluding Remarks

Considering these various effects of HDACs related to the epigenetic regulation of genes that are important for T_reg_ differentiation and maintenance, the development of a therapy setting including HDAC-specific inhibitors is a promising task. 

Based on the already established methods to generate and expand polyclonal, antigen-specific, or engineered T_regs_ ex vivo for adoptive cell therapy (for review see [52]), these can be treated with specific HDAC inhibitors to enhance Foxp3 expression, which will consequently induce and maintain an immunosuppressive T_reg_ phenotype. After these T_regs_ have been infused back, the limiting factor is the half-life of transferred T_regs_. This is especially important in patients with autoimmune diseases, where a permanent T_reg_-based immunosuppression is required. This holds true as well for patients following solid organ transplantation. Bone marrow transfer includes the risk of graft-versus-host disease, which exists temporarily and does not demand a very long T_reg_ life. However, in sepsis patients, the situation is completely different. Because here T_regs_ are mainly deleterious and contribute to an immunosuppressive state that is linked to an inappropriate immune response that finally causes a fatal outcome, the number of these cells should be reduced or their immunosuppressive phenotype should be immediately mitigated. This possibly can be achieved by HDAC inhibition, provoking Foxp3 destabilization or reduced expression. However, it should be taken into account, that, if the HDAC inhibitor is applied in an unspecific formulation to the sepsis patient, it will operate in all cells that express the corresponding HDAC. Therefore, putative side effects have to be carefully proven before. Moreover, a T_reg_ cell-specific HDAC-inhibitor formulation might be a chance to circumvent these side effect studies.

As discussed for autoimmune diseases, transplant rejection and sepsis, adequately altering the generation and number of T_regs_, i.e., increasing or decreasing their count, is associated with an improved outcome. To achieve this successfully, clinical trials are mandatory in the near future to clarify the role of epigenetics, especially during sepsis initiation and progression. Moreover, the development of HDAC-specific inhibitors is important to allow for the fine tuning of chromatin histone acetylation. Considering the expression and activity of the transcription factor Foxp3 as the main mediator for T_reg_ cell differentiation and function, its epigenetic modulation might be an appropriate target to reduce or enhance T_reg_ function according to the disease state.

## Figures and Tables

**Table 1:** Polymicrobial sepsis induced by a cecal ligation and puncture (CLP) operation in mice and rats increased regulatory T cell (T_reg_) count in the blood and spleen. Different mouse and rat strains have been used. The severity of the model is affected by the needle diameter, the number of punctures, and the ligation length [64,65]. (Ø, diameter; CLP, cecal ligation and puncture; f, female; G, gauge; m, male; MLN, mesenteric lymph nodes; and PC, peritoneal cavity.)

	Strain	Sex	Weight	Age	CLP				T_regs_	Ref.
			[g]	[weeks]	Ligation	Needle Ø	Perfo-Ration	Dura-tion	Organ	
**Mice**	BALB/c	m	-	8	immediately distal to the ileocecal valve		twice		↑ (spleen)	[67]
		m	20 ± 2	6–8	1/3, 2/3, 3/3	23G	single	24 h	↑ (spleen)	[68]
		m	20–25	8	50%	21G	once	15 d	↑ (spleen)	[69]
		m	18–22	-	50%	21G	twice	24 + 48 h	↑ (spleen)	[70]
		m	20 ± 1	6–8	below the ileocecal valve	18G	once	1/2/3/4 d	↑ (blood)	[71]
	C57BL/6	m	25	8	caecum ligated at its base	18G	twice		↓ (blood)	[72]
		f	-	6–8	50%	27G	twice	3/7 d	↑ (spleen)	[73]
		m	25–27	-	-	23G	-	48 h	↑ (spleen)	[74]
		m	25–35	-	50%	18G	twice	24 h	↑ (spleen)	[75]
		m	20–25	7–9	30% of its length	21G	twice	5 d	↑ (spleen)	[76]
		m	20–25	8–10	1.5 cm from the tip	22G	twice	20 h	↑ (spleen)	[77]
		m/f		6–8	below the ileocecal valve	21G	nine	24 h	↑ (PC, MLN)	[78]
		m	22–25	6–8		22G	once	3 d	↑ (spleen)	[79]
		m	20–25	-	75%	21G	twice	24 h	↑ (spleen)	[80]
		m	20–25	-	75%	21G	twice	24 h	↑ (spleen)	[81]
		m	22–30	8–10	below the ileocecal valve	22G	twice	24 h	↑ (spleen)	[82]
		m	25	8	at its base	21G	once	1/3 d	↑ (MLN)	[83]
		m	-	7	1 cm from the apex	18G	twice	16 h	↑ (spleen)	[84]
		f	-	8–12	30%	27G	once	24 + 48 h	↑ (spleen)	[85]
		m	-	8–12		22G	twice	24 h	↑ (spleen)	[86]
		m	-	6–8		22G	twice	30 h	↑ (spleen)	[87]
	FVB/N 9xNFAT luc	-	-	-	75%	21G	twice	24 h	↑ (spleen)	[88]
	ICR	m	30–35	6–8	50%	23G	twice	24 + 72 h	↑ (blood)	[89]
		m	27–29	-	at its distal site	20G	twice	26 h	↑ (spleen)	[90]
	NMRI		20–30	-	30%	27G	once	1/2/3 d	↑ (spleen)	[91]
**Rats**	Fischer	m	-	104	70% of its length	18G	twice	20 h	↑ (spleen)	[92]
	Wistar	m	250–300		50%	18G	twice	18 h	↓ (blood)	[93]
	Wistar Hannover	m	200–250	8	below the ileocecal valve	18G	twice	24 h	↑ (MLN)	[94]
	Sprague Dawley	m	350–400	-	distal ligation	18G	twice	3 d	↑ (blood)	[95]
	Sprague Dawley	m	320–350	-	distal ligation	18G	twice	3 d	↑ (spleen)	[96]
	Sprague Dawley	m	400–450	-	distal ligation	18G	twice	72 h	↑ (blood + spleen)	[97]

**Table 2:** HDAC isoforms expressed in T_regs_ promoting/attenuating their function. (C, colitis; CAT, cardiac allograft transplantation; CF, cystic fibrosis; C/JIA, collagen/juvenile-induced arthritis; CLP, cecal ligation and puncture; MCAO, mouse transient middle cerebral artery occlusion.)

Class	Isoform	Localization	Effect of HDAC Targeting	Specific HDACi	HDAC-Foxp3 Interaction	Models	Ref.
**I**	HDAC1	nucleus	↓	no	inhibits HDAC1	CAT, C	[152,153]
	HDAC2	nucleus	↑	in progress	associates with Foxp3	CAT, C	[153,154]
	HDAC3	nucleus/cytosol	↓	no	destabilizes Foxp3	CAT, C	[155]
	HDAC8	nucleus	↓	available	?	CAT	[155,156]
**IIa**	HDAC5	nucleus/cytosol	↓	no	?	CAT	[157]
	HDAC7	nucleus/cytosol	↓	no	forms a transcriptional complex with Foxp3	thymic positive and negative T cell selection	[158,159]
	HDAC9	nucleus/cytosol	↑	no	destabilizes Foxp3	C	[132,160,161]
**IIb**	HDAC6	nucleus/cytosol	↑	available	destabilizes Foxp3	CF, CIA, JIA, lupus prone mice	[160,162,163,164,165,166]
	HDAC10	nucleus/cytosol	↑	in progress	destabilizes Foxp3, re-presses Foxp3 transcription	CAT, C	[167,168,169]
**III**	SIRT1	nucleus	↑	available	destabilizes Foxp3	CLP, heterotrophic cardiac and ortho-tropic renal allo-graft, C	[87,126,160,169,170]
	SIRT2	cytosol	↑	no	destabilizes Foxp3	MCAO	[171]
	SIRT3	mito	↓	no	-	CAT	[172]
	SIRT4	mito	↑	no	inhibits Foxp3 expression	mouse spinal cord compression in-jury	[173]
**IV**	HDAC11	nucleus	↑	available	destabilizes Foxp3	CAT	[158]

## Abbreviations

Ø	diameter
Ac	acetylation
ALI	acute lung injury
ALS	amyolotrophic lateral sclerosis
AP1	activator protein 1
APC	antigen presenting cell
AS	ankylosis spondylitis
C	colitis
CAT	cardiac allograft transplantation
CD	cluster of differentiation
CF	cystic fibrosis
ChIP	chromatin immunoprecipitation
CIA	collagen-induced arthritis
CLP	cecal ligation and puncture
CNS	conserved non-coding region
CREB	cAMP-responsive element binding protein
CTLA4	cytotoxic T lymphocyte associated protein 4
ETS	E26-AMV virus oncogene cellular homologue
f	female
Foxp3	Forkhead-box-protein P3
g	gauge
H	histone
HAT	histone acetyltransferase
HDAC	histone deacetylase
HIF	hypoxia-inducible factor
HMGB1	high-mobility-group-protein B1
HP1	heterochromatin protein-1
i	in vitro
IBD	inflammatory bowel disease
ICOS	inducible T-cell costimulatory
Ikzf2	ICAROS family zinc finger 2
IPEX	immune dysregulation polyendocrinopathy and enteropathy
K	lysine
LPS	lipopolysaccharide
m	male
Me	methylation
MHC	major histocompatibility complex
MLN	mesenteric lymph nodes
MS	multiple sclerosis
NFAT	nuclear factor of activated T cells
NRa4	nuclear receptor 4a
Nt5e	ecto-5’-nucleotidase
OXPHOS	oxidative phosphorylation
p	periphery
PC	peritoneal cavity
PGC1α	PPARgamma coactivator alpha
PIAS	protein inhibitor of activated STAT
PSO	psoriasis
PTEN	phosphatase and tensin homologue
RA	rheumatoid arthritis
Pde3b	phosphodiesterase 3b
RAR	retinoid acid receptor
RUNX	Runt-related transcription factor 1
RXR	retinoid X receptor
SAHA	suberoylanilide hydroxamic acid
Satb1	special AT-rich sequence binding protein
SIRT	silent information regulator
Smad	small mothers against decapentaplegic
SP	single positive
SSc	systemic sclerosis
STAT	signal transducer and activator of transcription
sumo	small ubiquitin-like modifier
t	thymus
T1D	type 1 diabetes
TCR	T cell receptor
TIP60	HIV-Tat-interactive protein
TGF-β	transforming growth factor-beta
TLR	toll-like receptor
T_reg_	regulatory T cell
TSDR	T_reg_-cell specific region
